# An Approach to Greater Specificity for Glucocorticoids

**DOI:** 10.3389/fendo.2018.00076

**Published:** 2018-03-13

**Authors:** Carson C. Chow, S. Stoney Simons

**Affiliations:** ^1^Mathematical Biology Section, NIDDK/LBM, National Institutes of Health, Bethesda, MD, United States; ^2^Steroid Hormones Section, NIDDK/LERB, National Institutes of Health, Bethesda, MD, United States

**Keywords:** glucocorticoid specificity, antiglucocorticoids, selective glucocorticoid receptor modulators, induction, repression, mathematical model, site of cofactor action, kinetic mechanism of cofactor action

## Abstract

Glucocorticoid steroids are among the most prescribed drugs each year. Nonetheless, the many undesirable side effects, and lack of selectivity, restrict their greater usage. Research to increase glucocorticoid specificity has spanned many years. These efforts have been hampered by the ability of glucocorticoids to both induce and repress gene transcription and also by the lack of success in defining any predictable properties that control glucocorticoid specificity. Correlations of transcriptional specificity have been observed with changes in steroid structure, receptor and chromatin conformation, DNA sequence for receptor binding, and associated cofactors. However, none of these studies have progressed to the point of being able to offer guidance for increased specificity. We summarize here a mathematical theory that allows a novel and quantifiable approach to increase selectivity. The theory applies to all three major actions of glucocorticoid receptors: induction by agonists, induction by antagonists, and repression by agonists. Simple graphical analysis of competition assays involving any two factors (steroid, chemical, peptide, protein, DNA, etc.) yields information (1) about the kinetically described mechanism of action for each factor at that step where the factor acts in the overall reaction sequence and (2) about the relative position of that step where each factor acts. These two pieces of information uniquely provide direction for increasing the specificity of glucocorticoid action. Consideration of all three modes of action indicate that the most promising approach for increased specificity is to vary the concentrations of those cofactors/pharmaceuticals that act closest to the observed end point. The potential for selectivity is even greater when varying cofactors/pharmaceuticals in conjunction with a select class of antagonists.

## Introduction

Glucocorticoids are invaluable members of the arsenal of pharmacopeia used to treat numerous human pathologies. Their many uses include therapies for autoimmune disorders, allergies and asthma, adrenal insufficiencies, and various cancers. Their widespread usage stems from the fact that virtually every tissue type in the human body contains, and is responsive to, glucocorticoid receptors (GRs) [for recent review of glucocorticoid effects on 17 mouse tissues, see Ref. (1)]. Conversely, this ubiquity of sensitive cells means that it is very difficult to restrict glucocorticoid actions to one cell type, or location in the body, other than by external topical applications. Thus, systemic glucocorticoid administration is associated with various unwanted consequences or non-specific side effects. These side effects, or lack of specificity, of glucocorticoids severely limit their applications, especially for long-term treatments (2–4).

Glucocorticoid receptors are intracellular proteins found predominantly in the cytoplasm of cells and are associated with various chaperone proteins. Cellular entry of steroids/ligands occurs by passive diffusion, after which they bind to the ligand-binding domain of GRs. The resulting receptor–steroid complexes are then “activated” by a temperature-dependent step that results in the loss of chaperone proteins, increased nuclear binding, association with either DNA-bound proteins or biologically active DNA sequences (called hormone response elements), and recruitment of various transcriptional cofactors (e.g., chromatin remodeling factors, coactivators, corepressors, comodulators, etc.), thereby modifying the rates of gene expression to increase, or decrease, the levels of mRNA transcripts (5). Importantly, these assorted cofactors do not evoke the same responses during, or are even involved in all instances of, gene expression under GR regulation (6). This suggests that more selective gene expression by GR complexes during development, differentiation, and homeostasis may be achieved by careful control of these mechanistic components. Unfortunately, most of the steps and mechanistic details after GR–steroid complex binding to DNA are unknown, which greatly impedes efforts to minimize unwanted side-effects and to make glucocorticoid therapies more selective for specific genes and/or clinical outcomes.

Many attempts have been mounted over the years to improve glucocorticoid specificity and thereby expand their applications. The majority, and most promising, of these studies have concentrated on the binding of ligands to the cytosolic GR and the ability of the resulting GR–steroid complex to modify gene transcription. The first approach to be explored was variations in steroid structure. Unfortunately, single functional group modifications do not always convey the desired activity and the specific responses are almost as diverse as the steroid structures themselves (7–11). Furthermore, strong binding of the steroid does not assure agonist activity [compare Ref. (12, 13)]. More recently, it was hoped that the emergence of numerous transcriptional cofactors would offer a solution. Again, however, various responses defied any simple categorizations for increased selectivity (3, 14, 15).

Part of the challenge is the ability of glucocorticoids to cause both gene induction and gene repression. It was initially suspected that the undesired side effects of glucocorticoid repression result from the induction of other genes, and *vice versa*. However, this attractive hypothesis was discarded when the valuable anti-inflammatory actions of glucocorticoids were found to involve both gene induction and gene repression (4, 16). Furthermore, some genes are oppositely regulated in different cells (17). Finally, microarrays in two different cell lines (mouse mammary 3134 and mouse AtT-20) revealed no overarching generalities of induction vs. repression or the kinetics of each response (18).

Numerous other efforts to find phenomena that could be used to predict glucocorticoid selectivity have been similarly unsuccessful. These include GR–steroid complex binding to DNA (19, 20) or to glucocorticoid response element (GRE) sequences in the DNA closest to the responsive gene (21). Specific conformational changes in GR with the binding of different steroids appears to offer a promising correlation (22) but it is not yet possible to predict these changes for a given steroid. The same is true for chromatin remodeling by different GR–steroid complexes (23).

Selective glucocorticoid receptor modulators (SGRMs) offer yet another promising approach for steroids with greater specificity (10). SGRMs are compounds that have suboptimal gene regulatory properties and were previously classified as antagonists or antiglucocorticoids. This is because, as an antiglucocorticoid, they could reduce the total activity of a potent glucocorticoid, such as dexamethasone (Dex). However, most antiglucocorticoids, or SGRMs, are not pure antagonists, are unable to completely eliminate the activity of agonists such as Dex, and retain some partial agonist activity (PAA). For this reason, not all GR-activated, and not all GR-repressed, genes are affected by a given SGRM and more desirable combinations of affected genes are theoretically possible. However, it is not yet possible to predictably construct a SGRM with specific gene regulatory properties. Moreover, how antagonists are discovered is usually not clear (24, 25) and is more often than not by serendipity (26–28) than by directed efforts. A major impediment in designing SGRMs is that their mixture of partial agonist and partial antagonist activities is not constant in different cells (23, 24). Even more problematic is that the balance of agonist/antagonist activity for the same steroid/gene combination is not constant but varies with the experimental and cellular conditions (11, 29–31).

The above efforts to improve the selectivity of glucocorticoid responses have been inspired by the dramatic advances over the past 50 years in a mechanistic understanding of glucocorticoid steroid actions that are mediated by binding to the cytosolic GR. However, these advances are limited almost exclusively to the steps preceding the changes in initiation and rate of gene transcription. Because the subsequent steps are still largely undefined, no experimentally testable, conceptual framework has yet emerged with which to assess quantitatively possible improvements in glucocorticoid selectivity. One approach is to identify the gene(s) that is responsible for the desired (or undesirable) outcome and to study specificity in that system. For example, one might examine the induction of specific lung surfactant associated proteins (SP-A, SP-B, SP-C, and SP-D) in human fetal lung cells vs. other modified proteins (32). Unfortunately, this is often not possible because many desired glucocorticoid responses are very complex and not the result of changes in a single gene. For example, in studies of anti-inflammatory actions of Dex in human A549 lung cells, 2,766 genes were found to be regulated (33). However, even for single gene responses, a theory-based framework with which to rank specificity has not yet been advanced.

The purpose of this paper is to review an existing mathematical theory of gene transcription and show how it leads us to hypothesize a new method for quantifying increased glucocorticoid specificity. This theory has been used successfully over the past 8 years to predict how and where those factors that can modulate GR transcription properties actually exert their effect(s) in a sequence of gene expression steps (11, 34–44). The hypothesis is that greater specificity will be achieved when the site of action of the modulating factor is closer to the end of the numerous (albeit currently unidentified) steps involved in glucocorticoid-regulated expression of the target gene. The experimental approach is to look at the ability of any factor to influence the action not only of agonists and antagonists in gene induction but also of agonists in gene repression. For those factors having an effect, a graphical analysis of the results based on the mathematical theory can be used to determine where in the sequence of reactions, and how, the factor affects GR-regulated transcription. Here, we briefly summarize the theoretical basis for this graphical analysis and give examples of how the site and mechanism of action of various factors has been determined. With this approach, one can now screen for known and unknown factors that act near the last step of gene expression and then quantitatively determine their ability to increase the expression of the observed end product.

## Theory of Gene Transcription

### Model for GR-Regulated Gene Induction

The conventional model of glucocorticoid hormone action, and steroid hormone action in general, was formulated many years ago (45). However, even with more recent refinements (46, 47), the model remains qualitative and unable to make quantitative predictions. The need to have a quantitative model, with which one can compare predicted and experimental data, was our motivation for constructing a mathematically based, theoretical framework of glucocorticoid hormone action. Furthermore, an imperative of the mathematical model was to explain the well-known observation that, while the usual shape of the dose–response curve for glucocorticoid induced responses as a first-order Hill plot dose–response curve (FHDC) is invariant, the maximum activation (i.e., *A*_max_) and position of the curve (i.e., EC_50_) are not constant for a given inducing steroid (as predicted by the existing model of steroid hormone action) but rather vary in a systematic way with changing concentrations of numerous factors including GR itself (31, 35, 48–56). An understanding of this phenomenon is desirable because it is not limited to GRs but has been documented for all five classical steroid receptors (57–64). An additional critical observation is that the FHDC is not merely the result of averaging over a population of heterogeneous dose-response curves but rather that the probability of mRNA production in each cell obeys the same FHDC (65).

**Figure 1:**
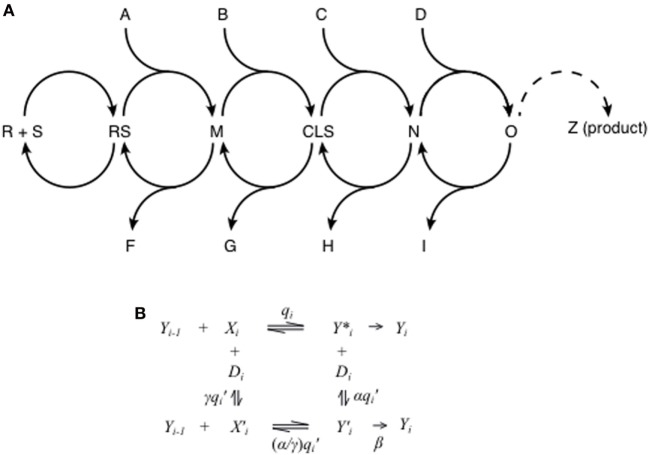
Theory of glucocorticoid-regulated gene expression. **(A)** Glucocorticoid induction and repression is assumed to obey a series of interconnected reaction steps. The first step is steroid (S) binding to receptor (R) to give the receptor–steroid complex (RS). The subsequent steps involve the possible input of various factors (A, B, C, D, etc.) to produce intermediates [M, concentration limiting step (CLS), N, O, etc.] and the possible loss of other factors (F, G, H, I, etc.). The dashed curve from intermediate “O” to the observed product “Z” is to indicate the presence of yet additional steps. One of the steps before “Z” is the CLS (see below in text), which may be anywhere but is shown, only for the purposes of illustration, as being between intermediates M and N. **(B)** In the mathematical model, each step in the sequence in **(A)** is represented by a set of enzymatic reactions where *Y_i_* is the reaction product of step *i*, *X_i_* is an accelerator, activating cofactor, or activator, and *D_i_* is a decelerator, inhibiting cofactor, or inhibitor. The labels on the reactions represent association constants for reversible reactions and reaction rates for non-reversible reactions. As in enzyme kinetics, we denote the case of α = 0 to be *competitive* inhibition, γ = 0 to be *uncompetitive* inhibition, α = γ to be *noncompetitive* inhibition, and α and γ both non-zero to be *mixed* inhibition. The case of β = 0 is called *linear* inhibition, and β > 0 is called *partial* inhibition. In general, computing the dose–response curve for such a reaction sequence would be analytically intractable. However, imposing the experimentally observed constraint that the dose–response curve has a Hill-coefficient of one yields a closed-form expression for the dose–response curve in terms of the parameters of all the reactions.

Many plots of response vs. added steroid in GR-mediated transactivation are described by a FHDC, which is the familiar Michaelis–Menten plot. This single fact places great constraints on the overall reaction sequence even though neither the number nor the identity of all of the steps between steroid (S) binding to receptor (R) and the appearance of the final gene product (Z) are known (Figure 1A). Despite this paucity of precise mechanistic information, we have previously shown that a reaction sequence composed of a series of interconnected, reversible complex-forming reactions is able to reproduce the observed FHDCs (40). Each of the arbitrarily many individual steps involves other molecular species that are, or are not, utilized (Figure 1A). Importantly, the location of where those species (A, B, C, D, etc.) act with respect to other species can be identified using the theory. During transcription, the local concentration of these species, plus those that are side products (F, G, H, I, etc.), will decrease, or increase, which would simultaneously affect the expression and/or abundance of other genes that are influenced by these species. It is known that small concentration differences in transcription factors, even as low as 10–15%, can alter the levels of gene transcription (53, 66–68). In this manner, each step of GR-regulated gene expression provides an opportunity and molecular mechanism for undesired side reactions. Therefore, one straightforward method for improving glucocorticoid specificity would be to identify those factors that act further downstream, and closer to the production of the desired gene product, to increase the flow of the reaction sequence from that location. A downstream factor would produce fewer changes than an upstream factor in the local concentrations of those species used by other genes (A, B C, D, F, G, H, I, etc.), which we hypothesize would increase the specificity of the glucocorticoid response.

So how is it possible to gain information about where, and by what mechanism, a factor acts in a reaction sequence that is composed of presently unknown steps? The answer is through observing how varying the concentration of factor affects the maximum response and the position of the dose–response curve, which can be accomplished using a simple graphical analysis (Table 1). It is known that changes in the concentration of various factors, including GR itself, can alter the position of the dose–response curve (31, 35, 48–56). By varying the amount of a single factor in GR-regulated gene expression, an examination of the appropriate plots yields information regarding how and where in the overall sequence of events that factor acts relative to a step called the concentration limiting step (CLS). Biochemically, the CLS is that step after which the concentration of the bound factors is much less than the free concentration of each factor. For example, it has been found that concentration of RNA pol II decreases as one goes from the 5′-end to the 3′-end of transcribed genes (69). Therefore, the CLS is analogous to, but not necessarily equivalent to, the rate-limiting step in enzyme kinetics. An important difference between the CLS of equilibrium systems and the rate-limiting step of enzyme kinetics is that while a factor present at low concentrations is a candidate for acting at the CLS, that factor does not have to act at the CLS. Specific details of the CLS are found in the mathematical equations (see Appendix) and its significance is seen in the descriptions of the various scenarios that can give rise to the numerous graphical plots (see Tables 1–4).

**Table 1:** Algorithms for single and double factor plots in GR-mediated gene induction.

(A) Single factor plots for factor F			

Plot parameters		Plot properties	Mechanistic conclusions
1/EC_50_ vs. F		Linear with 0 slope (i.e., does not change with F)	(1) F = A at concentration limiting step (CLS) (2) F = PN before CLS
		Linear with positive slope	(1) F = A not at CLS (2) F = U before or at CLS (3) F = PU before CLS
		Non-linear decreasing curve (concave-up)	(1) F = C (2) F = M before or at CLS
		Non-linear increasing curve (concave-down)	(1) F = LM or PM, before CLS (2) F = M at CLS

*A*_max_/EC_50_ vs. F		Linear; *y*-axis intercept = 0	(1) F = A before or at CLS
		Linear; *y*-axis intercept > 0	(1) F = A after CLS (2) F = PU before or at CLS
		Non-linear decreasing curve that approaches 0 for large F	F = C before or at CLS
		Non-linear decreasing curve that approaches positive value for large F	(1) F = PM or PN, before or at CLS (2) F = C after CLS
		Non-linear increasing curve	(1) F = PM or PN, before or at CLS

EC_50_/*A*_max_ vs. F		Linear with positive slope	(1) F = C before or at CLS

**(B) Two factor plots for factors F1 and F2**			

**Entry No.**	**Plot parameters**	**Plot properties**	**Mechanistic conclusions**

1	1/EC_50_ vs. F1 for different values of F2	Linear with 0 slope; curves do not change with F2	(1) F1 = PN before CLS and F2 = A at CLS

2		Linear with 0 slope; *y*-axis intercept increases with F2	(1) F1 = A at CLS and (a) F2 = A not at CLS (b) F2 = U or M at CLS (c) F2 = U or M or PN before CLS (2) F1 = PN before CLS (a) F2 = A not at CLS (b) F2 = U or M at CLS (c) F2 = U or M or PN, before CLS

3		Linear with 0 slope; *y*-axis intercept decreases with F2	(1) F1 = A at CLS and (a) F2 = C or M, before or at CLS (b) F2 = C after CLS

4		Linear; slope increases; curves do not change with F2	(1) F1 = A not at CLS and F2 = A at CLS (2) F1 = U before or at CLS and F2 = A at CLS (3) F1 = PU before CLS and F2 = A at CLS

5		Linear; slope increases with F2; lines intersect at F1 = 0	(1) F1 = A before CLS and (a) F2 = A after F1 and not at CLS (b) F2 = U or M, after F1 and at CLS (c) F2 = PU or PM, after F1 and before CLS

6		Linear; slope increases with F2; lines intersect at F1 < 0	(1) F1 = A before CLS and F2 = A before F1 (2) F1 = PU before CLS and F2 = A not at CLS (b) F2 = U after F1 and before or at CLS (3) F1 = U at CLS and F2 = A before F1 (4) F1 = A after CLS and F2 = A before F1 and F2 not at CLS

7		Linear; slope increases with F2; lines do not intersect at one point	(1) F1 = A before CLS and F2 = PU before or at F1 (2) F1 = PU before CLS and F2 = PU before CLS (3) F1 = U at CLS and F2 = PU before F1

8		Linear; slope decreases with F2; lines intersect at F1 = 0	(1) F1 = A before CLS and (a) F2 = C after F1 (b) F2 = M after F1 and before or at CLS (2) F1 = A after CLS; F2 = C at F1

9		Linear; slope decreases with F2; lines intersect at F1 < 0	(1) F1 = PU before CLS; and (a) F2 = C after F1 (b) M after F1 and before or at CLS (2) F1 = A after CLS and F2 = C at CLS

10		Linear; slope decreases with F2; lines do not intersect at one point	(1) F1 = A before CLS and F2 = C or M, before or at F1 (2) F1 = PU before CLS and F2 = C or M, before F1 (3) F1 = U at CLS and F2 = C or M, before F1 (4) F1 = A after CLS and F2 = C or M, before CLS

11		Linear; *y*-axis intercept increases with F2; lines never intersect (slopes of lines are the same)	(1) F1 = A after CLS and F2 = U at CLS (2) F1 = LU before CLS and (a) F2 = A before CLS and after F1 (b) F2 = U or M, before or at CLS after F1 (3) F1 = PU before CLS and F2 = LU before F1 (4) F1 = U at CLS and F2 = LU before F1

12		Linear; *y*-axis intercept decreases with F2; lines never intersect (slopes of lines are the same)	(1) F1 = A after CLS and F2 = C after CLS not at F1 (2) F1 = LU before CLS and F2 = C or M, before or at CLS and after F1 (3) F1 = U at CLS and F2 = C after CLS

13		Non-linear increasing, curves do not change with F2	(1) F1 = LM or PM, before CLS and F2 = A at CLS (2) F1 = M at CLS and F2 = A at CLS

14		Non-linear increasing curve; curve position increases with F2 (shape not preserved)	(1) F1 = LM before CLS and (a) F2 = A before F1 (b) F2 = PU or PM, before F1 (2) F1 = M at CLS and (a) F2 = A before F1 (b) F2 = PU or PM before F1 (3) F1 = PM before CLS and (a) F2 = A after F1 (b) F2 = U or M, after F1 at or before CLS

15		Non-linear increasing curve; curve position increases with F2 while preserving shape	(1) F1 = M before or at CLS and F2 = LU before F1

16		Non-linear increasing curve; curve position decreases with F2	(1) F1 = LM before CLS and F2 = C or M, before F1 (2) F1 = PM before CLS and (a) F2 = C or M, before CLS (b) F2 = C at or after CLS (3) F1 = M at CLS; F2 = C before or after CLS

17		Non-linear decreasing, curves do not change with F2	(1) F1 = C or M, before or at CLS and F2 = A at CLS (2) F1 = C after CLS and F2 = A at CLS

18		Non-linear decreasing curve; curve position increases with F2; curves go flat at F2 = 0	(1) F1 = C after CLS and F1 after F2 (a) F2 = A not at CLS (b) F2 = PM or PU, before CLS (2) F1 = C at CLS and (a) F2 = A before CLS (b) F2 = PU or PM, before CLS (3) F1 = C or M, before CLS and (a) F2 = A before F1 (b) F2 = PU before F1

19		Non-linear decreasing curve; curve position increases with F2; curves do not go flat at F2 = 0	(1) F1 = C after CLS and F1 before F2 (a) F2 = A not at CLS (b) F2 = PM or PU, before CLS (2) F1 = C at CLS and (a) F2 = A after CLS (3) F1 = C or M, before CLS and (a) F2 = A after CLS (b) F2 = U after F1 and before or at CLS (c) F2 = PU after F1

20		Non-linear decreasing curve; curve position increases with F2 while preserving shape	(1) F1 = C after CLS and (a) F2 = U at CLS (c) F2 = LU before CLS (2) F1 = C or M, before or at CLS and F2 = LU before F1

21		Non-linear decreasing curve; curve position decreases and gets flatter with F2; curve does not go flat for very large F2	(1) F1 = C before F2 (a) F2 = C (b) F2 = M before or at CLS (2) F1 = M before or at CLS, F1 before F2 (a) F2 = C (b) F2 = M before or at CLS

22		Non-linear decreasing curve; curve position decreases and gets flatter with F2; curve goes flat for very large F2	(1) F1 = C after F2 (a) F2 = C (b) F2 = M before or at CLS (2) F1 = M before or at CLS, F1 after F2 (a) F2 = C (b) F2 = M before or at CLS
		No equivalent to #20 exists for curve position decreases with F2	

23	*A*_max_/EC_50_ vs. F1 for different values of F2	Linear; slope increases with F2; lines intersect at *y* = 0, *y*-axis intercept = 0	(1) F1 = A at or before CLS and (a) F2 = A (b) F2 = PU or PM, before or at CLS

24		Linear; slope decreases with F2; lines intersect at *y* = 0, *y*-axis intercept = 0	(1) F1 = A at or before CLS and (a) F2 = C (b) F2 = M before or at CLS

25		Linear; slope and *y*-intercept increase with F2; lines intersect at *y* = 0, *y*-axis intercept > 0 (i.e., lines intersect at negative *x*)	(1) F1 = A after CLS and F2 = A or PU or M, before or at CLS (2) F1 = PU before or at CLS and (a) F2 = A (b) F2 = PU or M, before or at CLS

26		Linear; slope and *y*-axis intercept decrease with F2; lines intersect at *y* = 0, *y*-axis intercept > 0	(1) F1 = A after CLS and F2 = C or M, before or at CLS (2) F1 = PU before or at CLS and F2 = C or M, before or at CLS

27		Linear; slope increases and *y*-axis intercept fixed with F2; lines intersect at *y* > 0, *y*-axis intercept > 0	(1) F1 = A after CLS and F2 = A after CLS and after F1

28		Linear; slope and *y*-intercept increases with F2; lines intersect at *y* > 0, *y*-axis intercept > 0	(1) F1 = A after CLS and F2 = A after CLS and before F1

29		Linear; slope decreases and *y*-axis intercept fixed with F2; lines intersect at *y* > 0, *y*-axis intercept > 0	(1) F1 = A after CLS and F2 = C after CLS at or after F1

30		Linear; slope and *y*-intercept decrease with F2; lines intersect at *y* > 0, *y*-axis intercept > 0	(1) F1 = A after CLS and F2 = C after CLS and before F1

31		Non-linear; increasing curve; curve position increases with F2	(1) F1 = PN or PM, before or at CLS and (a) F2 = A (b) F2 = PU or PM, before or at CLS

32		Non-linear; increasing curve; curve position decreases with F2	(1) F1 = PN or PM, before or at CLS and (a) F2 = M or N, before or at CLS (b) F2 = C

33		Non-linear; decreasing curve that approaches 0 for large F1; curve position increases with F2 (see Note 10)	(1) F1 = C before or at CLS and (a) F2 = A (b) F2 = PU or PM, before or at CLS

34		Non-linear; decreasing curve that approaches positive value for large F1; curve position increases with F2 (see Note 10)	(1) F1 = PM or PN, before or at CLS and (a) F2 = A (b) F2 = PU or PM, before or at CLS (2) F1 = C after CLS and (a) F2 = A (b) F2 = PU or PM, before or at CLS

35		Non-linear; decreasing curve that approaches 0 for large F1; curve position decreases with F2 (see Note 10)	(1) F1 = C before or at CLS and (a) F2 = C (b) F2 = M or N, before or at CLS

36		Non-linear; decreasing curve that approaches positive value for large F1; curve position decreases with F2 (see Note 10)	(1) F1 = PM or PN, before or at CLS and (a) F2 = C (b) F2 = M or N, before or at CLS (2) F1 = C after CLS and (a) F2 = C (b) F2 = M or N, before or at CLS

37	EC_50_/*A*_max_ vs. F1 for different values of F2	Linear; slope increasing with F2	(1) F1 = C before or at CLS and (a) F2 = C (b) F2 = M or N, before or at CLS

38		Upward curving polynomial of degree *n*; position increasing with F2	(1) F1 = C before or at the CLS acting at *n* places (a) F2 = C (b) F2 = M or N, before or at CLS

39		Linear; slope decreasing with F2	(1) F1 = C before or at CLS and (a) F2 = A (b) PU or PM, before or at CLS

40		Upward curving polynomial of degree *n*; position decreasing with F2	(1) F1 = C before or at the CLS acting at *n* places (a) F2 = A (b) PU or PM, before or at CLS

41	EC_50_ vs. F1	Linear, no change with F2	(1) F1 = C and F2 = A at CLS

*Notation used is A = accelerator, C = competitive decelerator, U = uncompetitive decelerator, N = noncompetitive decelerator, M = mixed decelerator, L = linear decelerator, P = partial decelerator*.

*Plot properties (vs. F1) are listed by type of curve (e.g., linear or non-linear), how the curve changes as F2 changes, and the characteristics of the intersection points and intercepts of the family of curves. In mechanistic conclusions, multiple letter activities supersede single letter ones so that, for example, LN is also L. When multiple letter activities, followed by a comma, are listed before a description of the site of activity, that means that the site description applies to all of the activities. Of all of the possible scenarios, the most likely are completely described above, which represents over 74% of all combinations (44)*.

*Explanatory Notes*.

*(1) A “linear” plot by definition has a positive slope*.

*(2) Any apparently linear plot with a negative slope is non-linear by mathematical necessity*.

*(3) An essential accelerator by definition acts before or at the CLS*.

*(4) All references to *y* ≥ 0 refer to the situation when the total factor (endogenous plus transfected) equals 0*.

*(5) *y*-Intercept of *A*_max_/EC_50_ cannot be <0, while *y*-intercept of 1/EC_50_ is always ≥0*.

*(6) Curve position increases or decreases means the sum of the *y* coordinates of each curve increases or decreases*.

*(7) PU is like an accelerator acting after a local CLS and is indistinguishable from an accelerator acting after the last and global CLS*.

*(8) If a competitor acts at two steps before or at the CLS and the action at one step is weaker than the other, then the amount of upwards curvature in the EC_50_/*A*_max_ plot will be less pronounced*.

*(9) If low concentrations of a competitor appear (by a linear EC_50_/*A*_max_ plot) to act at one site before or at the CLS, while high concentrations of competitor appear to act at two sites (due to a quadratic fit of EC_50_/*A*_max_), this indicates the “emergence” of a second site of action at high concentrations of competitor*.

*(10) To determine whether or not a decreasing plot goes to 0 or a positive value, one looks at whether the plot of EC_50_/*A*_max_ vs. F1 is linear (in which case, the plot of *A*_max_/EC_50_ vs. F1 goes to 0) or whether the EC_50_/*A*_max_ vs. F1 plot can be linearized only after first subtract a constant plateau value from the *A*_max_/EC_50_ values and then calculating and plotting EC_50_/*A*_max_ (in which case, the plot of EC_50_/*A*_max_ vs. F1 goes to that plateau value)*.

**Table 2:** Mechanistic conclusions in GR-mediated gene repression by agonists based on dose–response parameter plots.

Entry	Plot properties of parameter vs. F	Mechanistic conclusions
1	*A*_max_ constant	1. F is any activity after or at GR and GR is A after concentration limiting step (CLS) 2. F is U after CLS and GR is any activity after F
2	*A*_max_ linear increasing through the origin	1. F is A at CLS
3	*A*_max_ non-linear increasing through the origin	1. F is A before CLS
4	*A*_max_ decreasing, approaches 0 for infinite F (1/*A*_max_ is linear increasing)	1. F is C or L before or at the CLS and GR is A after CLS 2. F is C or L before or at the CLS and GR is D before or at CLS
5	*A*_max_ and *A*_min_ non-linear increasing positive at true 0 of F	1. F is A after CLS and GR is A after F or GR is D 2. F is PU or PN before or at CLS 3. F is C after CLS and GR is A after F or GR is D
6	*A*_max_ non-linear decreasing does not approach 0 for infinite F	1. F is A after CLS and GR is A after F or GR is D 2. F is PU or PN before or at CLS 3. F is C after CLS and GR is A after F or GR is D
7	*A*_max_ increasing and *A*_min_ decreasing	1. F is A after CLS and GR is A or C, after F
8	*A*_min_ linear increasing through the origin	1. F is A at the CLS
9	*A*_min_ non-linear increasing through the origin	1. F is A before CLS
10	*A*_min_ positive at true 0 of F	1. F is A after CLS 2. F is PU or PN, before or at CLS
11	*A*_min_ decreasing, approaches 0 for infinite F (1/*A*_min_ linear increasing)	1. F is C, LU, or LN, before or at CLS
12	H of *A*_min_ < H of *A*_max_	1. F is A after CLS and GR is A after F 2. F is C or L, before CLS and GR is A after CLS
13	H of *A*_min_ > H of *A*_max_	1. F is A before CLS and GR is C or L, at F 2. F is A after CLS and GR is D before or at CLS 3. F is C after CLS and GR is A at F 4. F is C or L, at CLS and C < CLS and GR is A after CLS
14	H of 1/*A*_min_ < H of 1/*A*_max_	1. F is A after CLS and GR is A after F
15	H of 1/*A*_min_ > H of 1/*A*_max_	1. F is C after CLS and GR is A at F
16	H of 1/*A*_min_ = H of 1/*A*_max_	1. F is A after CLS and GR is D before or at CLS
17	IC_50_ constant	1. F is A at the CLS
18	IC_50_ increases	1. F is L before or at CLS and GR is A after CLS 2. F is C and GR is A after CLS 3. F is A before CLS and GR is C or L, before CLS
19	IC_50_ decreases	1. F is A before or after CLS and GR is A after CLS 2. F is PU or PN before CLS and GR is A after CLS 3. F is C or L, before CLS and GR is C or L, before CLS
20	*A*_max_ * IC_50_/*A*_min_ constant	1. Either F or GR acts before or at the CLS
21	*A*_max_ * IC_50_/*A*_min_ increases	1. F is C after CLS and GR is A after CLS 2. F is A after CLS and GR is D after CLS 3. F is D after CLS and GR is D after F
22	*A*_max_ * IC_50_/*A*_min_ decreases	1. F is A after CLS and GR is A after CLS 2. F is A after CLS and GR is D after F 3. GR is D after CLS and F is D after GR

*The predictions are derived by examining the formulas for these parameters as shown in Table S2 of Chow et al. (37). F means factor, GR means steroid–receptor complex, A means accelerator, D means any type of decelerator, C means competitive decelerator, U means uncompetitive decelerator, N means noncompetitive decelerator, L means linear decelerator, and P means partial decelerator. H is the concentration for the half-maximum of the dose–response parameter. This table represents sufficient conditions and is not complete. Note that two cofactors cannot be of the same type at the same step, GR will not repress gene expression if it is an A before the CLS, and the *A*_min_ graphs allow GR to act anywhere as a D or as an A after the CLS*.

**Table 3:** Mechanistic conclusions re GR-mediated gene induction by antagonists, or selective glucocorticoid receptor modulators, for changes in added receptor with downstream difference in equilibrium constant at location *d*.

Plot properties with increasing receptor	Mechanistic conclusions
PAA saturates at 100%	Step *d* is after steroid–receptor binding but before or at the concentration limiting step (CLS)
PAA increases to a maximal value less than 100%	1. Equilibrium constant of antagonist at step *d* is less than that of agonist at step *d* and *d* is not at CLS 2. Equilibrium constant of antagonist is greater than agonist and *d* is after the CLS
PAA decreases to a minimal value greater than 100%	1. Equilibrium constant of antagonist is greater than agonist and *d* is not at CLS 2. Equilibrium constant of antagonist is less than agonist and *d* is after CLS
PP increases	Equilibrium constant of antagonist is less than agonist
PP decreases	Equilibrium constant of antagonist is greater than agonist
PAA/PP does not change	Always true

**Table 4:** Predictions re GR-mediated gene induction by antagonists, or selective glucocorticoid receptor modulators, for changes in added accelerator at location *j* after the CLS with downstream difference in binding affinity of reaction components at location *d*.

Plot properties with increasing accelerator	Mechanistic conclusions
PAA saturates to 100%	*d* is before or at *j*
PAA does not saturate to 100%	*d* is after *j*
Saturated PAA less than 100%	1. Equilibrium constant of antagonist at step *d* is less than that of agonist at step *d* and *d* is after *j*. Product is mostly derived from steps after *d*. 2. Equilibrium constant of antagonist is greater than agonist and *d* is after *j*. Product is mostly derived from steps before *d*.
Saturated PAA greater than 100%	1. Equilibrium constant of antagonist is greater than agonist and *d* is after *j*. Product is mostly derived from steps after *d*. 2. Equilibrium constant of antagonist is less than agonist and *d* is after *j*. Product is mostly derived from steps before *d*.
PAA increases as linear-fractional function to maximum of 100%	Equilibrium constant of antagonist is less than agonist and *d* is before or at concentration limiting step (CLS)
PAA decreases as linear-fractional function to minimum of 100%	Equilibrium constant of antagonist is greater than agonist and *d* is before or at CLS
PAA is not a linear-fractional function	*d* is after CLS
PP increases	Equilibrium constant of antagonist is less than agonist
PP decreases	Equilibrium constant of antagonist is greater than agonist
PAA/PP does not change	*d* is before or at CLS
PAA/PP increases	Equilibrium constant of antagonist is greater than agonist and *d* is after CLS
PAA/PP decreases	Equilibrium constant of antagonist is less than agonist and *d* is after CLS
EC_50_ of PAA as function of receptor number decreases	*d* is before or at CLS

Even more information is obtained when the concentrations of two factors are systematically varied. Now, the graphical analysis also uncovers the ordering of the action, as opposed to binding, of the two factors relative to each other and to the CLS (Table 1). Thus, by comparing the results of a series of factors (A, B, and C), two at a time, one can deduce the order of all three and the CLS, e.g., B < C < CLS < A (where < denotes before). From such results, one would predict that changing concentrations of factor A would yield greater specificity in the transactivation of the gene being examined than would changes in factor B.

The importance of these competition assays in informing on that step in the reaction sequence where a factor acts cannot be over stressed as most methodologies indicate only where a factor binds. However, it is well known that when and where a factor binds is often unrelated to the site of factor action. p300 is recruited by added androgen to the androgen-responsive elements of the TMPRSS2 and FKBP5 genes but is required for androgen induction of only the former gene (70). RNA polymerase II is frequently found bound to pausing sites at about 50-bp downstream from the start of transcription of the induced gene but is not engaged in elongation until a later time (71–73). For glucocorticoid steroids, the action of agonist steroids appears to be very soon after their binding to receptor because nuclear accumulation and DNA binding of GR are two of the required, early steps in GR-regulated transcription and they occur in cells only after steroid binding (45, 74). This means that it is highly unlikely that agonist steroids of different structures will be able to confer as much increased specificity as changes in a factor that acts much closer to the end of the reaction sequence.

### Theoretical Basis of Graphical Analysis

Before describing the method of graphical analysis that permits the relative ordering of pairs of factors in a given transactivation experiment, it is important to briefly explain the theoretical basis for the analysis. The theory is specified in detail in papers on induction (38, 40), repression (37), and antagonists (11). In those papers, we show that a system of biochemical reactions does not generally lead to a FHDC. However, if the reactions obey detailed balance (i.e., if each step is independently reversible), and if multimeric complexes involving more than two species are only formed at or after the CLS, then a FHDC is possible. Given the models of multimeric factor complexes in transcription (47, 75), this suggests that the CLS may be at a relatively early position in the reaction sequence. This balance does not necessarily imply that all reactions in transcription are reversible, just that their outcomes can be effectively modeled by a set of reversible reactions [e.g., see Ref. (37)]. For such a system of reactions, an explicit formula of the dose–response curve in terms of the dissociation or equilibrium constants and the factor concentrations can be written down for an arbitrary number of reaction steps and species (Figure 1A). The mathematical details are provided in the Section “Appendix.” The formula is rendered useful when probing a fixed number of factors because the (mostly unknown) parameters in the dose–response curve can be collapsed into a small set of “effective” parameters acting before and after the CLS that completely characterize the dose–response curve and can then be fit to the data. *In lieu* of a direct model fit, properties of the factors and where they act with respect to each other and the CLS can be deduced using a graphical analysis (similar to Lineweaver–Burk plots of biochemistry) because the dose–response curve changes in qualitatively distinct ways for each configuration, which are manifested as the properties of certain graphs.

Implicit in the theory are new chemical kinetic and mechanistically descriptive alternatives to label the actions of a modulatory factor. The traditional terms of “coactivator” and “corepressor” consider only the ability of factors to increase and decrease, respectively, the total amount of gene product without reference to mechanism. In fact, classical chemical kinetics already tell us that the final product can increase due to two opposite actions of a factor: (a) to activate or accelerate an intermediate step or (b) to inhibit or decelerate a currently utilized step, thereby allowing a more productive but otherwise avoided step to be followed (76, 77). For example, each individual step in Figure 1A can be described as a set of reactions depicted in Figure 1B, where Y*_i_*_−1_ is the product of the preceding step and Y*_i_ and *Y* ′*_i_* are intermediates of the steps involving X*_i_* (without or with D*_i_* respectively) leading to the product Y*_i_*, which initiates the next “step” in the overall reaction sequence. X*_i_* is always an accelerator while D*_i_* is always a decelerator. When elevated amounts of X*_i_* increase the rate of reaction to Y*_i_, and thus the amount of Y*_i_, then Y*_i_* also increases. However, if Y*_i_ is much less efficient than *Y* ′*_i_* in producing Y*_i_*, then X*_i_*′s action as an accelerator to increase the amount of Y*_i_ can actually decrease the amount of Y*_i_* and the amount of final, observed product. In a similar manner, higher levels of the decelerator D*_i_* can decrease or increase Y*_i_* concentrations. Thus, under the traditional terminology, X*_i_* (and D*_i_*) would, under different conditions, appear to be either a coactivator or a corepressor, even though its site and mechanism of action is unchanged (37). A further refinement is that while D*_i_* can increase or decrease the net output from any site before or after the CLS, X*_i_* can do so only at positions after the CLS. Before the CLS, X*_i_* only increases the net output. Therefore, we have adopted the terms “accelerator” and “decelerator,” which describe how a given species alters the reaction kinetics at that step where each species acts (34). In the language of chemical enzyme kinetics, an accelerator acts like an activator while a decelerator acts like an inhibitor. Like inhibitors in enzyme kinetics, decelerators can be competitive, uncompetitive, noncompetitive, mixed, linear, or partial (Table 1). In this context, the action of GR itself can also be described as an accelerator or decelerator. In fact, the theory argues, and experimental data confirms (37), that the distinction between steroid-mediated induction and repression is simply how and where GR acts (e.g., GR is an accelerator before the CLS for induction and an accelerator after the CLS for repression). This action could vary between genes or possibly even different alleles of the same gene. Finally, the theory automatically incorporates the action of antagonists and predicts that their action is the same as steroids except that one or more of the equilibrium constants are perturbed. The theory unifies induction, repression, and antagonist activity into a universal framework.

It should be noted that the above development of the mathematical theory proceeded without restrictions on the identity of the components. The only requirements are that the final dose–response curve display a FHDC. This means that the theory is general and not limited to GR action or even steroid hormone action. Any inducible biological response that yields a FHDC can be examined using these same methods. For the purposes of this review, however, we will confine ourselves to examples with steroid receptors and almost exclusively to GR.

A prediction of the theory of GR action was that the binding of GR to DNA does not occur as preformed dimers, as described in the then accepted models of steroid hormone action (46, 47). Instead, the above model requires that DNA-bound GR dimer formation occurs as a result of the step-wise binding of GR monomers. In examining GR binding to DNA in detail, we found that four GR dimerization defective mutants, including a new double mutant, still cause steroid induced transactivation with the same FHDC-type plots as wild-type GR, although with higher EC_50_ (40). These results strongly argue that dimerization of GR is not mandatory for DNA binding, as required by our theory.

### Analysis of Agonists in GR-Mediated Gene Induction

We first tested if the model could account for the changes in both maximal total activity (*A*_max_) and position of the dose–response curve (EC_50_) when the target cells were cotransfected with increasing amounts of a GR cofactor, Ubc9 (78, 79). Multiple concentrations of GR and Ubc9, each with multiple concentrations of inducing steroid, were used to generate a series of dose–response curves. The agreement of the theoretical curves with the experimental data was very good (40). Direct mathematical fits of the predicted dose–response curves were then used to determine that Ubc9 was functioning as an activator [later called accelerator (34)] at a step after the CLS, thereby confirming and extending previous conclusions (78, 79).

**Figure 2:**
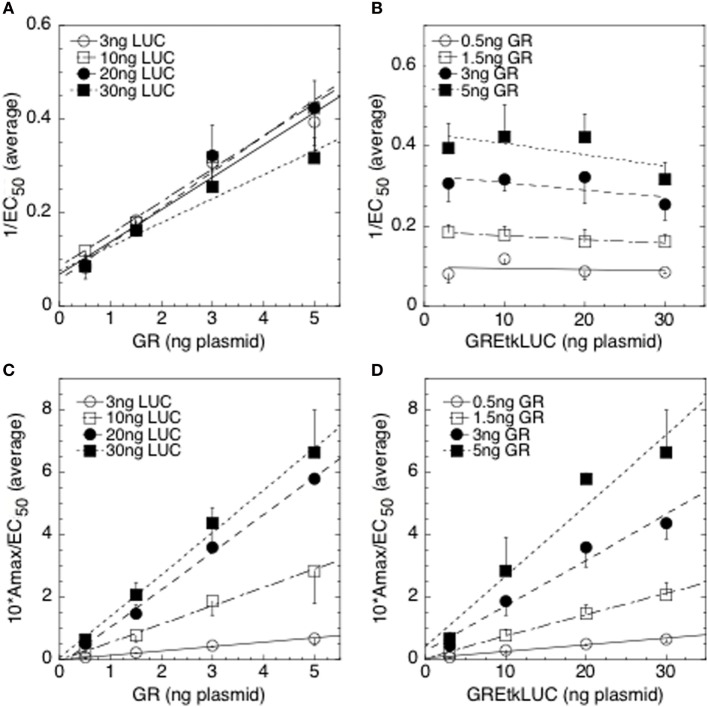
Competition assay with glucocorticoid receptor (GR) and GREtkLUC during GR-mediated induction in 293 cells. All combinations of four concentrations each of GR and GREtkLUC plasmids for a total of 16 sets, all in triplicate, were used to cotransfect 293 cells, which were then treated with ethanol, or three subsaturating concentrations of Dex in ethanol, before determining the amounts of induced luciferase. Exact fits of these data to a first-order Hill plot yielded the *A*_max_ and EC_50_ for each combination [for details, see Ref. (34)]. Graphs of 1/EC_50_ vs. GR **(A)**, 1/EC_50_ vs. GREtkLUC **(B)**, *A*_max_/EC_50_ vs. GR **(C)**, and *A*_max_/EC_50_ vs. GREtkLUC **(D)** are the averages of three independent experiments (34).

A much easier method for obtaining the same information was subsequently developed that involves analyzing a series of graphs (Table 1) instead of fitting curves (38). Briefly, one first determines the *A*_max_ and EC_50_ for each of the combination of the factors being examined. We define *A*_max_ as the maximum amount of induced activity, which is determined from first-order Hill plot fits of the activity vs. steroid concentration with each combination of the factors. The fits of these plots are usually very good with (*R*^2^ ≥ 0.99) (41, 44). One must then determine, usually by Western blots, whether the expression of each of the factors is, or is not, linear over the range of transfected cDNA used. To do this, one plots the amount of factor detected vs. transfected factor cDNA. If the expression is not linear, then the expression is “linearized” by using the observed amount of expressed factor (arbitrary units) as opposed to amount of transfected cDNA in the subsequent plots of 1/EC_50_, *A*_max_/EC_50_, EC_50_/*A*_max_, and EC_50_ vs. the “linear” amounts of factor 1 vs. factor 2 and of factor 2 vs. factor 1. Finally, the amount of endogenous factor must be added to the linearized added amount of factor. Therefore, what is plotted on the *x*-axis is the total amount (exogenous plus endogenous) of factor 1 or factor 2. To make plots combining the results of several experiments, one can simply average the data (e.g., Figure 2). Alternatively, the data can be normalized, usually to the lowest amount of each of the two factors (41). In both situations, the average values ± error are then plotted. The appearances of these plots (linear, curved, etc., under “Plot Properties”) are then compared to a table of the most common mechanistic behaviors of factors in gene expression (under “Mechanistic Conclusions”) (Table 1). Each of the plots in Table 1 can be generated by several different mechanisms (1a, 2b, etc.), which differ in the mode and usually the sites of action of the two factors relative to the CLS. Typically, one will examine the plots of 1/EC_50_ and *A*_max_/EC_50_ (or of 1/EC_50_, *A*_max_/EC_50_, and EC_50_/*A*_max_) for factor 1 with varying factor 2 and *vice versa* for a total of 4 (or 6) types of plots. Often, the appearance of a given type of plot can be compatible with more than one of the “Plot Properties” of Table 1, in which case all possibilities must be considered initially. However, many of the multiple mechanistic explanations are not represented in each of the 4 (or 6) types of plots and these can be immediately discarded. Some mechanistic descriptions are more specific, which leads to the elimination of yet other possible explanations. By further examination of the remaining candidate mechanisms for internal consistency, one is invariably able to identify the one mechanism of factor 1 and of factor 2 that is compatible with all 4 (or 6) plots. The Text Box 1 describes in detail the steps used to interpret any group of plots.

Text Box 1 **Methodology for using tables to interpret graphs of experimental results.**

The interpretation of any set of graphs of experimental results proceeds as follows, using the graphs of Figure 2 as an example. First, one identifies those graphical descriptions of Table 1 that most closely describe each of the graphs of Figure 2. For 1/EC_50_ vs. GR, entry #4 appears to be the closest descriptor but entry #8 is also possible. For 1/EC_50_ vs. GREtkLUC, the best match is with entry #2 but #18 might also be appropriate. For *A*_max_/EC_50_ vs. GR, the only reasonable entry is #23 while #23, and possibly #25, could describe *A*_max_/EC_50_ vs. GREtkLUC. One then makes a chart of all of the mechanistic conclusions for GR, and for GREtkLUC, that are given for each of the identified entries for each graph. In an effort to find one consistent set of mechanistic conclusions for GR and for GREtkLUC in all of the graphs, one notes that there is only one reasonable entry for *A*_max_/EC_50_ vs. GR, which states that GR is an accelerator (A) before or at the CLS and GREtkLUC is either an A acting anywhere or a PU or PM before or at the CLS. Therefore, all other mechanistic conclusions in the chart can be eliminated. Importantly, the conclusions of entry #8 to explain 1/EC_50_ vs. GR, and of entry 18 to explain 1/EC_50_ vs. GREtkLUC, can all be discarded because none of them have GREtkLUC acting as an A. Furthermore, the only explanation in entry #23 for *A*_max_/EC_50_ vs. GR is that GR is an A acting before or at the CLS. Therefore, all remaining mechanistic conclusions in the above chart that have GR being anything other than an A before or at the CLS can be removed. For both graphs of 1/EC_50_, one is left with GR being an A not at the CLS and GREtkLUC being an A at the CLS. When this conclusion is combined with the remaining possibilities for the graphs of *A*_max_/EC_50_, the only mechanistic conclusion that is compatible with all four graphs is that GR acts as an A before the CLS and GREtkLUC acts as an A at the CLS.

Once one starts assigning the site and mechanism of action of numerous factors in different cell lines, two major questions arise. The first is whether the results with transfected synthetic reporter genes are a reasonable model for an endogenous, native gene. The second is whether the location of the CLS is fixed or changes with the composition of the added factors. With regard to the first question, it should be remembered that the mathematical model applies only to those genes for which the GR-regulated expression displays a FHDC. For many glucocorticoid inducible genes, the dose–response curve is not a FHDC (18, 80). While such gene induction behaviors immediately tell us that mechanisms other than that of the above mathematical model are operative, one cannot use it to uncover what they may be. Examining the induction of endogenous genes requires mRNA analysis, which is much more laborious than quantifying the output of a luciferase reporter gene. However, it can be done. When the action of two different cofactors, TIF2 and sSMRT, on GR induction of the endogenous TAT gene in U2OS cells was examined, it was found that they were the same as seen for GR induction of the synthetic reporter gene (GREtkLUC) in the same U2OS cells. We therefore conclude that the actions of cofactors in GR-regulated gene induction is very similar, if not identical, to those employed with endogenous genes displaying a FHDC.

The second question, whether the location of the CLS is fixed, is important given the large number of species known to contribute to receptor-steroid control of gene expression (81) and to the importance of the CLS as a possible common reference point in descriptions of the site of factor actions. As seen in Table 1, most of the mechanistic conclusions are relative to the CLS. There is no *a priori* reason to believe that the site of any factor action, including the CLS, would remain the same under all conditions. However, if the position, not to mention the physical identity, of the CLS is constant, this would make the CLS an invaluable standard reference point, about which one could use overlapping competition assays to order the actions of all other factors, even when the factor acting at the CLS is not one of the two factors being examined. Therefore, it is critical to determine what the CLS is, at least in one system, and then whether the CLS is invariant in other systems. When such a study was conducted, it was found that that the reporter gene (GREtkLUC or MMTVLUC) always acted at the CLS in 4 different cell lines with 1 accelerator and 5 different decelerators for a total of 12 different reaction conditions (34). Therefore, it is reasonable to conclude that the reporter gene (i.e., the GRE) always acts as an accelerator at the CLS, that the CLS is invariant in its position in the total sequence of events, and that all other factors can be organized relative to the CLS even when the reporter gene is not directly examined in the reaction.

**Figure 3:**
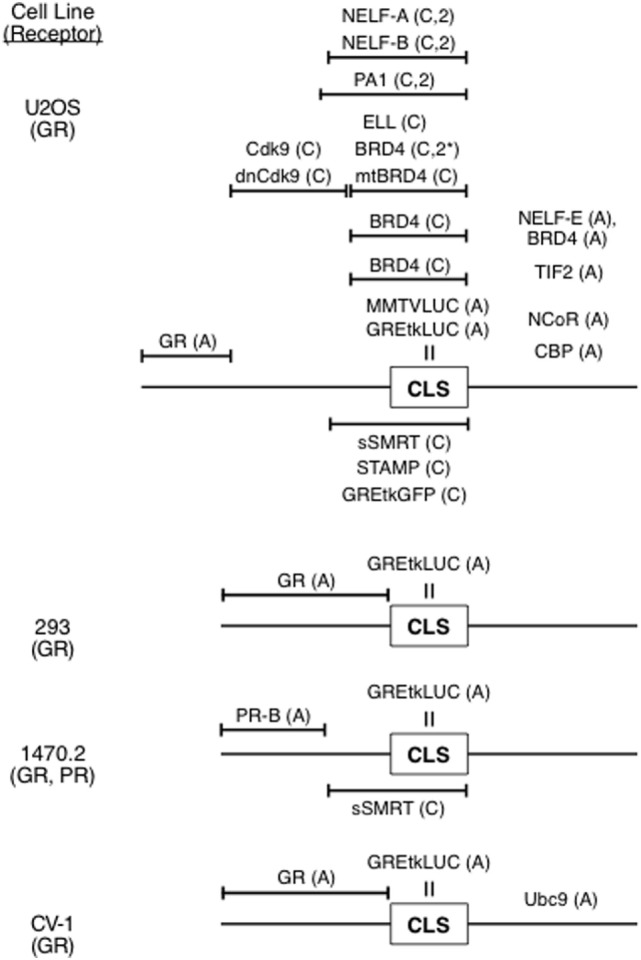
Ordering of factors in reaction scheme for induction of luciferase activity from synthetic reporter (GREtkLUC, MMTVLUC) by steroid-bound receptor [glucocorticoid receptor (GR), progesterone receptor (PR)]. The position of the concentration limiting step (CLS), which is the site of action of the reporter, and positions of action of various factors relative to the CLS and other factors are indicated. Abbreviations: A, accelerator; C, competitive decelerator; C,2, competitive decelerator at two sites; C,2*, competitive decelerator at two sites for BRD4 only with relatively high concentrations of CDK9.

With the identification and constancy of the CLS, it has been possible to combine the data of multiple GR transactivation experiments (34, 41, 43) to indicate how and where numerous factors exert their effects (Figure 3). With this approach, factors that previously were not thought to modify GR-regulated gene transcription have been readily identified and their site and mechanism of action determined. Examples of such novel modulators include PA1 (Pax2 transactivation domain interaction protein-associated protein 1) (43), each of the four components of the NELF complex (39), the kinase Cdk9 (44), and BRD4, which is a kinase involved in the initial steps of gene induction (41). This information is currently not available by any other method. It is therefore relevant that the sites of action of Cdk9, BRD4, NELF complex, NELF-E, and TIF2 are consistent with the biochemical data for these factors (41).

NCoR is commonly considered a corepressor (82, 83). Nevertheless, the above graphical methodology for determining the kinetically defined mechanism of action of a factor finds that NCoR functions as an accelerator in GR-controlled gene induction (34). Importantly, there is no contradiction in a corepressor (defined by the factor’s effect on product level) functioning as an accelerator (defined by the mechanism at the site where the factor acts). As it was described earlier, if NCoR accelerates what is otherwise a relatively unproductive step after the CLS, which gives a low yield of the next intermediate in the overall reaction scheme, then the net output in the presence of NCoR would be lower and NCoR would be classified as a corepressor.

### Analysis of Agonists in GR-mediated Gene Repression

It has been estimated that 2.6 million people in the US over 20 years old took oral glucocorticoids over the 10-year period of 1999–2008, with a mean usage duration of 4.4 years (84). While not reported, it is probable that the majority of prescriptions were for anti-inflammation. While it is now realized that the anti-inflammatory actions of glucocorticoids involve both gene induction and gene repression (4, 16), many of the important actions involve gene repression, where glucocorticoids decrease the expression of various genes. This response is opposite to that of glucocorticoids in gene induction and thus greatly complicates discussions of factor activity. For example, many cofactors are classified as either coactivators or corepressors based solely upon their ability to increase or decrease, respectively, the level of steroid-mediated gene expression (47, 83). However, these definitions were established for situations were the agonist Dex increased gene expression. Clearly, these definitions will not be applicable when Dex decreases gene expression, as it does for gene repression.

Another issue in gene repression is that the concentration of glucocorticoid required for half-maximal response is not constant but, as in gene induction, varies with the experimental conditions (30, 53, 85–87). There is no explanation in the current models of GR-mediated repression for this behavior, which obviously directly affects steroid doses in anti-inflammatory applications. Our theory and analysis tool could help to resolve and understand the above issues and to obtain a more precise and quantitative understanding of the mechanisms of gene repression. In particular, we could classify the activity of all factors by their kinetically defined mechanism at the affected step in the reaction sequence (i.e., accelerator or one of six types of decelerator) and also obtain information regarding where in the overall sequence a given factor is acting. None of this information is currently available from existing methodologies.

We first verified that the dose–response curve was a FHDC. This was found to be true for the frequently used system of Dex repression of PMA induction of an AP1LUC reporter gene in U2OS.rGR cells (37), which are U2OS cells containing stably transfected rat GR (88). The most information about GR-mediated gene repression was obtained in a competition assay of two different factors. Table 2 was constructed to relate the molecular mechanisms to the appearance of the various graphs. As with gene induction, the amount of factor plotted on the *x*-axis represents the total amount (exogenous and endogenous) of factor after correction for any non-linear expression from the transfected cDNA. The graphs for gene repression, which differ from those used in Table 1 for gene induction, involve the maximal activity with no added steroid (*A*_max_), the minimum value of activity with saturating steroid concentrations (*A*_min_), and the concentration of steroid for half-maximal suppression (IC_50_). These parameters will always be for a first-order Hill plot of the factor concentrations. Thus, the factor concentration for half-maximal response (H) provides additional information about the factor’s action. The mechanistic conclusions for each set of graphs is then determined using the same logic as presented in the Text Box 1 for gene induction but now with the entries of Table 2.

The mathematical model for gene repression was then used to examine the action of four factors [the reporter gene, the p160 coactivator TIF2, and two pharmaceutical chemicals: NU6027 and phenanthroline (see below)] in GR repression of PMA induction of the synthetic AP1LUC reporter gene in U2OS.rGR cells. All four factors were found to act as accelerators (37) *via* precisely the same mechanism and at the same site of action as was observed for each of the factors in GR transactivation assays (35, 38). Therefore, we conclude that while TIF2, NU6027, and phenanthroline augment the total response in gene induction and decrease the response in gene repression, their mechanism, and site of action does not change. In both systems, TIF2, NU6027, and phenanthroline each act as an accelerator after the CLS. This critical information greatly simplifies efforts to increase the specificity of glucocorticoids: it suggests that the mechanisms of factor action do not change in GR-regulated induction and repression and only one process need be considered in both situations. Finally, manipulation of factor concentration in induction vs. repression is predicted to simultaneously affect both pathways.

The net responses to glucocorticoids in gene induction and repression are diametrically opposed. Therefore, it was not clear that the CLS would be the same in the two systems. By simultaneously varying the reporter (AP1LUC) and NU6027 or phenanthroline, it was found that AP1LUC acts as an accelerator at the CLS (37). Thus so far, in both gene induction and gene repression with GR, the reporter (through the agonist response element) always acts at the CLS as an accelerator.

**Figure 4:**
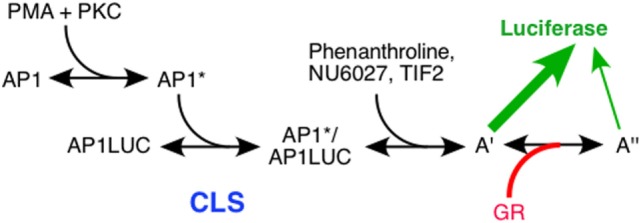
Flow chart of actions of factors in glucocorticoid receptor (GR)-regulated gene repression. Schematic diagram of PMA induction of Luciferase activity from synthetic reporter (AP1LUC) by AP1 that is repressed by steroid-bound receptor (GR). The position of the concentration limiting step (CLS), and sites of action of TIF2, NU6027, phenanthroline, and GR are indicated. A′ and A″ represent unknown, post-CLS steps, each of which can lead to Luciferase activity but the efficiency from A″ is much less than A′ [from Ref. (37)].

If the factors (TIF2, NU6027, and phenanthroline) and the reporters (GREtkLUC and AP1LUC) have the same site and mode of action in gene repression and induction, the only parameter that can change is GR. Usually one measures the response to changes of a factor to determine the action of that factor. Unfortunately, this was not technically possible for GR because the vigorous repression required for accurate graphical analyses was observable only with the high levels of stably transfected GR. Fortunately, close scrutiny of the equations for the theory of repression indicated that one can deduce the position and mechanism of GR action from assorted graphical properties with respect to another cofactor. Using this type of analysis, the only internally consistent explanation of the results for varying amounts of AP1LUC and TIF2 was that GR acts as an accelerator after TIF2, which is an accelerator after the CLS. The conclusions from experiments with AP1LUC and NU6027, or phenanthroline, were again that GR acts (with NU6027), or consistent with GR acting (for phenanthroline), as an accelerator after each chemical (37). These deductions are reminiscent of GR repressing TNFα induction of IL-8 gene expression by working after transcription initiation (89). In each case, the organization of the individual steps determines whether an accelerator exerting its effects after the CLS increases or decreases the total gene activity. We propose that decreased AP1LUC activity is due to GR acting as an accelerator to augment a less productive step downstream of PMA-mediated induction, which would decrease the amount of LUC expression (Figure 4). These results further suggest that GR retains the identical mechanism of action as an accelerator when interacting with other factors in induction and repression. Therefore, we conclude that the only difference between GR-mediated gene repression and gene induction is that the position of GR action changes to a site that is further downstream than any other factor yet identified: the mechanism of GR action, though, remains the same and is that of an accelerator.

### Analysis of Antagonists, or SGRMs, in GR-Mediated Gene Induction

Antisteroids or antagonists are defined as ligands that compete with agonist steroid hormones for the binding to cognate receptor proteins to reduce the biological activity of the agonist. However, it soon became apparent that most antisteroids retain significant amounts of agonist activity with some target genes. For this reason, such antagonists were actually partial agonists and were called “selective receptor modulators” or “selective glucocorticoid receptor modulators” for GR-binding ligands (90). The amount of residual agonist activity of the SGRM with a given reporter gene is often referred to as the PAA. If concentrations of a given SGRM sufficient to saturate the binding of the intended receptor protein give 30% of the maximal activity seen with saturating concentrations of a full agonist, that SGRM is said to have 30% PAA.

Before the advent of SGRMs, when limited reporter genes were available and various antisteroids were found to possess different amounts of PAA, it was presumed that the value of the PAA was an invariant property of that antagonist (91). The discovery of SGRMs for estrogen receptors, such as tamoxifen and raloxifene, which displayed PAAs that varied with the gene examined, discredited this hypothesis but raised the hope that SGRMs could be found with a desirable mix of genes that were, and were not, induced. Nonetheless, it was commonly believed that, for each SGRM, at least the PAA value with a given reporter gene in a specific cell was constant. Subsequent detailed studies revealed, though, that this concept too was invalid. In particular, the relative concentration of various transcription factors and cofactors, including coactivators, corepressors, and the receptors themselves, were found capable of acting like a rheostat to vary the PAA from, in some cases, close to 0 to almost 100% (15, 31, 48, 79, 92–94). One might intuitively expect that the PAA would remain constant under such conditions. For example, take a partial agonist that gives 30% PAA. With the addition of a coactivator, which by definition increases the absolute activity of the agonist, one might expect that the PAA of the antisteroid would be increased proportionately to give 30% of the now larger value of the agonist steroid, so that the PAA does not change. However, this is not the case. The addition of a coactivator usually augments the activity of the antisteroid more than that of the agonist so that the PAA increases (15, 31, 48, 79, 92–94). While this variability was puzzling, it suggested that the biological responses to SGRMs were malleable. If the underlying determinants of such modulation could be elucidated, then SGRMs might become very selective in their actions and thus extremely valuable in human treatments.

The above described theory of GR action, which can model antagonist activity without any modifications, indicates that differences in binding affinity of each SGRM alone cannot account for a difference in PAAs. Rather, the variations in activities between SGRMs require differences at a second biochemical step that is downstream from receptor–antagonist binding. The position of this second step can be probed with added factors. As with the initial model, the expanded model provides graphical methods for locating the position of this second step, relative to the CLS (Tables 3 and 4) (11). A new term that is introduced by this model is the partial potency (PP). The PP is the ratio of the potency, measured in terms of EC_50_, of the partial agonist, or SGRM, to the control agonist, such as Dex. The mechanistic conclusions for each set of graphs is then determined using the same logic as mentioned in the Text Box 1 for gene induction but now with the entries of Tables 3 and 4.

If a factor is known to increase the activity of a particular antagonist, then the theory predicts that all antagonists that converge to the same PAA value with large amounts of that factor exert their action at a single, common mechanistic step. This provides a novel means of classifying antagonists. Therefore, to the first approximation, a given class of antagonists would be predicted to have the same spectrum of specificity for gene expression. This description of SGRMs by the expanded mathematical model is more nuanced than the popular explanation that the altered steroid structure of antisteroids perturbs the topology of the resulting receptor–steroid complex from that seen with agonists, thereby acting like a binary switch, with corepressors replacing the coactivators that associate with agonist-bound receptors (95, 96). The newer model for antiglucocorticoid action also provides a logical approach for identifying more selective SGRMs by searching for those classes of antagonists that act furthest downstream of the CLS.

**Figure 5:**
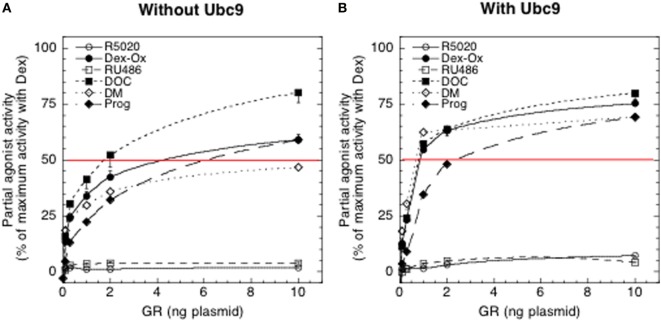
Effect of changing concentrations of glucocorticoid receptor (GR) without (A) and with (B) Ubc9 on partial agonist activity (PAA) of antiglucocorticoids. Experiments were conducted with 1 μM antisteroid. Luciferase activities were determined and the PAA of each steroid was calculated relative to 1 μM Dex under the same conditions. The values of four independent experiments were averaged and plotted ± SEM. The thin horizontal line at 50% is only for reference [from Ref. (11)].

The expanded model was applied to study the effect of increasing GR plasmid, with and without added Ubc9, on the PAA of six different antiglucocorticoids: [11-deoxycorticosterone (DOC), progesterone (Prog), R5020, RU486, dexamethasone oxetanone (Dex-Ox), and dexamethasone 21-mesylate (DM)] (Figure 5). While both accelerators and decelerators are accommodated by the model, only the accelerator Ubc9 has been directly examined to date. Analysis of the PAA values as a function of receptor number was reasonably well fit by a first-order Hill plot both without and with added Ubc9, as predicted by the theory. Additional analysis indicated that differences in PAA do not result from differences in binding affinity alone but result from unequal biochemical actions downstream from receptor–agonist binding. Using the above prediction that all SGRMs that converge to the same PAA with large amounts of a factor differ from agonists in their action at a single mechanistic step, the six antiglucocorticoids were grouped into three classes. The first class is that of covalently bound DM. Covalently bound DM has been found to have no agonist activity in two systems (13, 29). However, the amount of covalent labeling varies with the cell system (29). We strongly suspect that the PAA ± Ubc9 of DM plateaus at a value less than the ≈100% value that is extrapolated for the second class of DOC, Dex-Ox, and Prog because approximately 30% of the GR–DM complexes are covalently bound and inactive. Thus, DOC, Dex-Ox, Prog, and non-covalently bound DM would all give 100% PAA with sufficient GR and/or Ubc9. This behavior indicates that the second step that is modified by these antagonists occurs immediately after the step influenced by Dex binding to GR. Thus, it is unlikely that a member of this second class of SGRMs will display much more selectivity than full agonists that act at the preceding step.

The third class is composed of RU486 and R5020. The PAA of both did increase with Ubc9 but was still dramatically lower than 100%. If PAA never reaches 100% with excess Ubc9 (Figure 5B), then the analysis suggests that the position of the affected step for each steroid is after that of Ubc9 (11), which is after the CLS (Figure 3). However, we cannot completely eliminate the possibility that the tertiary structures of the receptor–steroid complexes of RU486 and R5020 may be less influenced by, or have a lower affinity for, cofactors than the other antisteroids. In fact, there is less binding of the accelerator/coactivator GRIP1/TIF2 to DNA-bound GR–RU486 complexes than to GR–Dex complexes (15). Nonetheless, this method of analyzing SGRMs argues that greater selectivity may be achieved with those steroids that show low activity with added GR and/or coactivator like Ubc9 because they can exert effects after both the CLS and a downstream acting factor.

A popular explanation for the mechanism of SGRM action is that the altered steroid structure of antisteroids perturbs the topology of the resulting receptor–steroid complex from that seen with agonists, thereby acting like a binary switch, with corepressors replacing the coactivators that associate with agonist-bound receptors (95, 96). Our theory offers an alternative explanation. There are two steps influenced by SGRMs. The first step is SGRM binding to GR. With that group of antiglucocorticoids that can be forced to yield 100% full agonist activity, the second step occurs immediately after the site of GR–Dex complex action and could involve cofactor binding. However, this cofactor binding does not occur in a binary switch or all-or-nothing fashion. Rather, the binding of factors like “coactivators” and “corepressors” would be subject to equilibrium considerations to act like a rheostat, as has been previous documented (92). However, it is those SGRMs that do not yield 100% agonist activity, even with large amounts of factor, that are the most interesting. This is because it is likely that the second step now occurs after the CLS and after the accelerator being varied in the same experiment. The nature of this altered, downstream step is not yet known, and may depend on the SGRM, but is again subject to equilibrium reactions. The modification could be related to changes in the topology of the steroid–receptor complex.

Finally, these data suggest that very few, if any, reversibly binding ligands may be a full antagonist (with no PAA) under all conditions. We predict that the ability of each antisteroid to modulate a step in the downstream interactions will not be conserved but will depend to some extent upon the steroid structure. At least part of this may derive from changes at the affected step in the affinity of interacting factors, which can differ with steroid structure. This means that, under whole cell and whole organ conditions with limited variations in cofactor concentrations, the achievable PAA values will vary with antisteroid structure, even when the same mechanistic step is affected. It also indicates that the current view of antisteroids as “defective” agonists should be replaced by one where antisteroids selectively reduce the efficacy of individual downstream steps. This, in turn, provides opportunities to fine-tune gene expression in a manner that may be relatively unique for each combination of antagonist steroid, cofactor, gene, and cell type. GR-regulated gene transactivation of a synthetic reporter gene was used in the above study. However, given the similar response of synthetic and endogenous genes in previous reports (6, 38), it is anticipated that the present theory will be transferable to the study of antisteroids with endogenous genes, as long as the dose–response curve is described by a FHDC. Such information with endogenous genes would be invaluable in formulating more selective endocrine therapies with antisteroids.

### High-Throughput Screening for Identification of New Cofactors

The above methods have been used to identify and characterize several new factors in GR-regulated gene expression: PA1 (43), CDK9 (44), and BRD4 and NELF-E (41). These factors join the more than 300 factors reported to participate in steroid-regulated gene transactivation (81). It is likely that many more factors exist, some of which will act very far downstream and thus offer the possibility of greatly improving the specificity of GR action. Therefore, what is needed is a rapid method to identify new factors, especially small molecular weight chemicals that are easier to produce and administer to patients than biologics such as proteins, antibodies, cDNAs, and siRNAs. These requirements are nicely met by robotic high-throughput screening. One has to modify the setup to involve dose–response curves instead of single dose samples, which does dramatically increase the number of wells required to analyze each chemical. Nevertheless, the amount of information obtained from such a procedure argues that it is worth the extra effort.

A high-throughput assay with 1,280 compounds yielded many new pharmaceuticals that alter the *A*_max_ and EC_50_ of GR transactivation (35). The top 10 modifiers of GR activities from the high-throughput assay (positive discovery rate = 0.78%) were then subjected to the above conventional competition assay in a different cell line (U2OS vs. 293 cells) for two purposes: first, to see if the properties of the chemicals were maintained in a different system and second, determine their mode and site of action. Of the 10 compounds, 4 are accelerators after the CLS, 5 are competitive decelerators at or before the CLS, and 1 is a competitive decelerator at 2 sites at or before the CLS. Importantly, the changes in *A*_max_ in the two different cell lines were qualitatively identical in 60% of the cases and possibly as high as 90%. The agreement increased to 90% when the modulation of EC_50_ was monitored (35). Interestingly, camptothecin causes qualitatively identical changes in *A*_max_ and EC_50_ in yet another cell line (CV-1) with high amounts of transfected GR and exogenous GREtkLUC reporter (97). Collectively, these results suggest that the current high-throughput assay can be used to identify chemicals that will be similarly active in various cell lines and will be of great use in identifying additional chemicals that modulate GR induction properties. The above competition assay can then be used not only to confirm the results of the high-throughput assay but also to identify those compounds that act after the CLS and would be predicted to impart greater specificity to GR transactivation and repression.

## Discussion

All approaches to date to increase the specificity of clinically powerful glucocorticoid therapies have suffered from an inability to discern those quantitative features needed to achieve the proposed changes, be it for steroid structure, receptor and chromatin conformation, DNA sequence for receptor binding, associated cofactors, etc. We have described an approach that is based on the fact that the dose–response curves for many examples of GR regulation of gene expression are first-order Hill plots not only with synthetic reporter genes in tissue culture but also with numerous endogenous genes. This is true for gene induction by agonist steroids and SGRMs, or antiglucocorticoids, and for gene repression by glucocorticoids. However, this new approach looks not at specific structural modifications but rather at the reaction kinetics, which are analyzed by simple graphical methods. The results from competition assays with any two factors, A and B, are that one can determine not only the kinetically defined mechanism of action of each factor at that step in the overall reaction sequence where the factor acts, as opposed to binds, but also the placement of each factor in the reaction sequence relative to each other and the CLS, which the above results indicate is where the DNA sequence of the regulated gene exerts its effect (note: the DNA sequence of the gene is certainly required, but is not a kinetically limiting factor, for the many subsequent steps). Thus, in a competition assay of A and B for GR induction of a reporter gene, one might find that B acts as a competitive decelerator before the CLS while A acts as an accelerator after the CLS. By conducting paired competition assays with various factors, one can identify that factor that acts closest to the observed end point.

It is reasonable to propose that increased specificity of glucocorticoid action will result from varying the concentration of that factor acting closest to the desired end point. This is because varying the concentration of a factor that acts many steps before the end point is going to modify the levels of all other species utilized in subsequent steps to produce the desired end product. This, in turn, will affect the local concentrations of each of these species. Most, if not all, of these species would be expected to be common components of gene transcription in general. Therefore, local concentration changes in these common species will affect the control mechanisms for other gene products, resulting in alterations of the levels of off-target proteins, i.e., unwanted side reactions of the glucocorticoid steroid. The only method currently available to determine the position of action of any species is the above described competition assays that are based on the mathematical model of steroid hormone action.

Glucocorticoid therapies often involve agonists or antagonists in gene induction and gene repression. Of the four possible combinations, the only one that we have not directly examined is antagonists, or SGRMs, in gene repression. Nevertheless, the results from the three other combinations now allow one to narrow the options for increasing glucocorticoid specificity. In the transactivation of gene by agonists, GR is found to act before the CLS, the reporter (agonist response element) acts at CLS, and several protein cofactors act after CLS in multiple systems (Figure 3) (34, 38, 41, 44). Thus, it is likely that the same factors will be found to act at the same positions in other systems induced by GR. In transactivation by SGRMs, the variations in activities between different antagonists were shown to be influenced by two parameters. The first was changing concentrations of various accelerators (15, 31, 48, 79, 92–94). The second was that class of SGRMs that always show activities significantly less than 100% agonist activity, even with increased amounts of accelerator (11). With the one accelerator that was examined, Ubc9, this group of SGRMs appears to act downstream of both the CLS and the accelerator, thus potentially making these SGRMs the most downstream factors observed so far in transactivation. In gene repression by agonist steroids, everything was the same as in gene induction except that GR now acts further downstream of the CLS than any other factor yet tested. While this suggests that GR would be an excellent target for increased glucocorticoid specificity in gene repression, GR’s position at the beginning of the sequence in gene induction (34, 39, 43, 44) eliminates it from further consideration. On the other hand, cofactors that act as accelerators downstream of the CLS in gene repression also act as accelerators downstream of the CLS in gene induction and thus are promising candidates for increased selectivity. Finally, those antiglucocorticoids, or SGRMs, such as RU486 and R5020 are very attractive for two reasons. First, generally only a fraction of those genes induced by a full agonist are also induced by SGRMs (24, 25, 27). Thus, SGRMs start off with some increased selectivity because the portfolio of genes affected by SGRMs is expected to be less than for full agonists. Second, those SGRMs that have significantly less than full agonist activity, even in the presence of added accelerators, appear to alter a step downstream both of the CLS and the accelerator (11). Thus, such SGRMs are predicted to have the two desired properties for increased selectivity: a reduced number of genes that are affected at a step that is much closer to the observed response.

More than 300 factors have been identified (81). In addition, high-throughput screening of pharmaceuticals rapidly identifies additional accelerators acting after the CLS (35). Such compounds are highly desirable (1) because they are much easier to administer than proteinaceous cofactors and (2) because there is often pre-existing data regarding human tolerances, thereby allowing one to quickly eliminate from further study those with known toxicity issues.

In conclusion, we have presented arguments supporting a rational approach for increasing the selectivity of glucocorticoid therapies. This approach relies on a relatively new competition assay system that identifies the kinetically defined mode and site of action of any factor in GR-mediated gene induction or repression by agonists and in gene induction by SGRMs. We conclude that the most profitable avenue of research for greater GR specificity is on varying the concentrations those factors/pharmaceuticals acting closest to the observed assay end point. Particularly attractive is the combination of accelerators that are pharmaceuticals with those SGRMs that always give less than 100% of full agonist activity because a reduced set of genes are modulated by easily administered compounds at a site that is close to the end of the reaction sequence. This combination will also be much easier to evaluate for human safety, thereby increasing the probability of generating clinically useful protocols. Finally, it should be noted that the same methodology can be employed to increase the specificity of any other ligand-regulated modifier of gene transcription that displays FHDC plots.

## Author Contributions

SS and CC jointly conceived and wrote this paper.

## Conflict of Interest Statement

The authors declare that the research was conducted in the absence of any commercial or financial relationships that could be construed as a potential conflict of interest.

## Appendix

### Mathematical Theory of Gene Transcription

Consider a reaction sequence of steps (labeled from 0 to *n*), where each step has the form in Figure 1B. In each step, a product *Y* binds with an accelerator *X* to make a new product. The step can be influenced by a decelerator *D* that can decrease (or in some cases increase) the new product. The *q* coefficients are equilibrium (inverse dissociation) constants, and α, β, and γ are nonnegative constants that determine the type of enzymatic inhibition at that step (e.g., competitive, uncompetitive, and non-competitive). Each reaction is assumed to obey detailed balance so that, at equilibrium, they satisfy the conditions $Y_{i}=Y_{i}^{*}+\beta Y_{i}^{\prime }$, $Y_{i}^{\prime }=\left(\alpha_{i}∕\gamma_{i}\right)q_{i}X_{i}^{\prime }Y_{i-1}$, $Y_{i}^{*}=q_{i}X_{i}Y_{i-1}$, $X_{i}^{\prime }=\gamma_{i}q_{i}^{\prime }D_{i}X_{i}$, and $Y_{i}^{\prime }=\alpha_{i}q_{i}^{\prime }D_{i}Y_{i}^{*}$, which imply $Y_{i}^{\prime }=\alpha_{i}q_{i}q_{i}^{\prime }D_{i}X_{i}Y_{i-1}$ and $Y_{i}=q_{i}X_{i}\left(1+\alpha_{i}\beta_{i}q_{i}^{\prime }D_{i}\right)Y_{i-1}$. For simplicity, we let the species name and its concentration be denoted by the same symbol. Before the CLS, the molecular species obey the conservation conditions $X_{i}+X_{i}^{\prime }+Y_{i}^{*}+Y_{i}^{\prime }=X_{i}^{T}$, which leads to the linear-fractional function (first-order Hill plot)

$$Y_{i}=\frac{\nu_{i}Y_{i-1}}{1+w_{i}Y_{i-1}}$$

where $\nu_{i}=\frac{q_{i}X_{i}^{T}\left(1+\alpha_{i}\beta_{i}q_{i}^{\prime }D_{i}\right)}{1+\gamma_{i}q_{i}^{\prime }D_{i}}$ and $w_{i}=\frac{q_{i}\left(1+\alpha_{i}q_{i}^{\prime }D_{i}\right)}{1+\gamma_{i}q_{i}^{\prime }D_{i}}$. At the CLS, the conservation condition is $X_{i}+X_{i}^{\prime }+Y_{i}^{*}+Y_{i}^{\prime }+\sum_{k=\text{cls}+1}^{n}Y_{k}=X_{i}^{T}$, which leads to the same *v*_i_ but $w_{i}=\frac{q_{i}\left(\sum_{k=i}^{n}\varepsilon_{k}\prod_{j=i+i}^{k}\nu_{j}+\alpha_{i}q_{i}^{\prime }D_{i}\right)}{1+\gamma_{\text{cls}}q_{\text{cls}}^{\prime }D_{\text{cls}}}$. After the CLS, the conservation condition is $X_{i}+X_{i}^{\prime }=X_{i}^{T}$, which implies that α*_i_* = 0 and β*_i_* = 0 (i.e., there is only competitive inhibition after the CLS) and $\nu_{i}=\frac{q_{i}X_{i}^{T}}{1+\gamma_{i}q_{i}^{\prime }D_{i}}$ and *w_i_* = 0. These conservation conditions imply that large multimeric complexes can only exist in significant concentrations at or after the CLS. The linear-fractional function is preserved under function composition (i.e., forms a group) and thus the product at any step is also a linear-fractional function of any previous step. Hence, for *m* steps, where *m* ≤ cls, the relationship between product *Y_m_* and *Y*_0_ is still linear-fractional

$$Y_{m}=\frac{V_{1}^{m}Y_{0}}{1+W_{1}^{m}Y_{0}}$$

where $V_{b}^{m}=\prod_{i=b}^{m}v_{i}$ and $W_{b}^{m}=\sum_{i=b}^{m}w_{i}V_{b}^{i-1}$. After the CLS, all the products have the same denominator. This implies that all steps after the CLS can lead directly to the gene product while preserving the position of the dose–response curve. Thus, the final gene product *P* can be written as

$$P=\frac{\Gamma V_{\text{1}}^{\text{cls}}Y_{0}}{1+W_{1}^{\text{cls}}Y_{0}}$$

where the positive constant $\Gamma =\sum_{k=\text{cls}}^{n}\;a_{k-\text{cls}}V_{\text{cls}+1}^{k}$ represents the sum over steps after the CLS. Note that *P* is a linear-fractional function of *Y*_0_ as well as every accelerator and decelerator concentration. In previous work, we assumed that for gene induction, steroid was *Y*_0_ and GR was *Y*_1_ (38, 40), while for repression GR was either a decelerator or an accelerator after the CLS (37). However, for both induction and repression, GR could possibly act as an accelerator or decelerator anywhere and the dose–response curve would still be a first-order Hill plot. We thus see that the difference between induction and repression is simply where GR acts and the baseline induction rate carries information about where GR acts. Although the dose–response formula depends on a large number of unknown parameters, we note that they collapse into effective *V* and *W* parameters, which uniquely specify *A*_max_ and EC_50_ for induction and *A*_min_, *A*_max_, and IC_50_ for repression. If we are to probe the mechanism and location of action of other parameters, we simply make them explicit in the formulas for *V* and *W*. This is aided by the decomposition formula $W_{a}^{b}=W_{a}^{c}+V_{a}^{c}W_{c+1}^{b}$. For example, if we were interested in some step *j* before the CLS, we merely write *V*^cls^ as *B*_1_*v*_j_ and *W*^cls^ as *B*_2_ + *B*_3_*w_j_*, where the *B* parameters are positive constants that capture the collective action of all the other (hidden) reactions. *V* and *W* for all steps before the CLS have the same functional form, which differ from those at steps acting at the CLS and after the CLS. The same expansion can be derived for more factors. For any finite number of factors, there are only a finite number of possible functional forms for combinations of accelerators or decelerators acting in relative order to each other and the CLS. These have been enumerated elsewhere (37, 38, 40). By observing how *V* and *W* change experimentally as the factor concentrations change, we can deduce which functional form best accounts for the data and thus infer the mechanism and relative location of action of that factor. This analysis can be accomplished by plotting graphs of *A*_max_ and EC_50_ vs. the factor concentration for induction and *A*_min_, *A*_max_, and IC_50_ vs. the factor concentrations for repression and then consulting tables of formulas or using verbal summaries as in Tables 1–4.

Finally, we incorporate the action of antagonists by hypothesizing that the rate constants (i.e., equilibrium or dissociation constants) of one or more of the reactions differ between different antagonists. The details are worked out in Chow et al. (11). We note that in Chow et al. we assumed that the steroid acted in the first reaction for gene induction and thus steroid–receptor binding affinity cannot affect the PAA. However, if steroid acts at another step other than the first, then the binding affinity could affect PAA.
